# Germination Response of *Datura stramonium* L. to Different pH and Salinity Levels under Different Temperature Conditions

**DOI:** 10.3390/plants11233259

**Published:** 2022-11-27

**Authors:** Nebojša Nikolić, Valentina Šoštarčić, Laura Pismarović, Maja Šćepanović, Roberta Masin

**Affiliations:** 1 Department of Agronomy, Food, Natural Resources, Animals and Environment, University of Padova, 35020 Legnaro, PD, Italy; 2 Department of Weed Science, Faculty of Agriculture, University of Zagreb, Svetošimunska 25, 10 000 Zagreb, Croatia

**Keywords:** jimsonweed, abiotic stress, germination, salinity, pH

## Abstract

Weeds can be one of the most severe threats to crop production, especially when they are widespread and highly adaptable. Part of the adaptive strategy of plants is the ability to germinate in different conditions. Germination is the first developmental phase of plant life and is fundamental for its establishment. In this work, the germination of two populations of *Datura stramonium* L. at two different sites in Croatia (one cropped, the other non-agricultural) was tested under a wide range of salinity stress, 4, 8, 12, and 16 dS/m, and pH stress, values 1–9, at two temperature ranges of 15–25 °C and 18–30 °C. The results show that this species can tolerate high salinity, with a high number of seeds germinating, even under the highest level of saline stress and especially at higher temperatures: 21.7% of seeds germinated at 15–25 °C and 51.2% at 18–30 °C. *D. stramonium* also appears to be quite acid tolerant, with a significant reduction in germination only at pH 2, and no germination only at pH 1. Germination was always higher at higher temperatures, independently of abiotic stress. Although there were some differences between the two populations in the final germination percentages, they were similar in their responses to the abiotic stresses.

## 1. Introduction

Weeds are a major concern in agricultural plant production, due to their competition with crops for water, nutrients, light, and space. In addition, they can be an obstacle to mechanical harvesting and hosts for pests, while some are toxic and can be harmful to humans and animals [1,2]. All of these attributes are species-specific, which highlights the importance of studying individual weed species in order to better understand the specific problems they may pose to agricultural production and to seek solutions [3,4]. One of the weed species that can cause great harm to both agriculture and human and animal health is *Datura stramonium* (L.) [5,6,7]. This is an annual species belonging to the Solanaceae family growing up to two metres in height. It can be branched, has large leaves shading the soil, and has easily recognizable white trumpet-like flowers, from which are formed fruits—spiny capsules—containing up to 30,000 dark, kidney-shaped seeds [5,8]. The origin of this species is disputable and there are many different citations in the literature. However, most papers claim its probable origins to be the tropical regions of Central and South America and it is usually found in warm-temperate and subtropical regions. *D. stramonium* has become a cosmopolitan weed and can be found in warm regions of North, Central, and South America, Europe, Asia, Africa, and New Zealand, and in more than 100 countries. It prefers open fertile fields but can also survive in sandy soils. It can also be found in abandoned, ruderal sites from sea level to more than 2000 m a.s.l. [5,6,9,10]. The plants are aggressive colonizers of agricultural fields and compete fiercely with summer crops, such as maize (*Zea mays* L.) and soybean (*Glycine max* L. [Merr.]), in many parts of the world causing substantial losses [11,12,13,14]. In addition to the problems it causes in agriculture, this species is also known to contain tropane alkaloids, mainly scopolamine, hyoscyamine, proline, atropine, etc. [15,16]. There are many reports of the harmful effects of this species if ingested by humans or animals, with potentially fatal outcomes caused by the aforementioned alkaloids [17,18]. Ingestion may be involuntary, as when parts of the plant are mixed with other plants; for example, *D. stramonium* seeds can contaminate different legumes and enter the diet or they can contaminate cereals that are subsequently ground to flour and used in different meal preparations [19,20,21,22]. Ingestion may also be voluntary, as this plant was, for centuries, used as a drug due to its hallucinogenic effects [19,23]. Unfortunately, this practice continues even today, adding to the notoriety of this plant and necessitating stricter controls [21,23,24,25]. Due to the health-related issues surrounding this plant, far more studies have been made on its chemical and medicinal properties than on the problems it can cause in agriculture. *D. stramonium* has a high invasive potential that is likely to increase with climate change, as reported in a study on the growth of this species when exposed to rising CO_2_ [26]. In addition to increases in atmospheric CO_2_ concentrations, there are many other factors associated with climate change to consider which may affect the spread of weed species, such as rising temperatures and variations in the amounts and patterns of precipitation, increased soil salinisation, and changes to soil pH levels. High levels of soil salinity induce stress in cultivated crops, negatively affecting their growth and development [27,28]. Soil salinisation is one of the consequences of low rainfall, the weathering of native rocks, rising sea levels, and poor agricultural practices, such as irrigation with high-salinity water. Much of this is a result of climate change and rising temperatures [29,30,31,32]. About 20% of arable land is estimated to be saline or salt-affected and this could reach 50% by 2050 [33]. Other studies indicate that rising temperatures in northern Africa and the Mediterranean may increase soil salinity [30]. Alterations to rainfall patterns as a result of climate change may also alter the soil pH [34], which can have significant effects on plant growth and development, as it influences substance mobility and the availability of mineral elements and nutrients. These effects may be positive or negative depending on a number of variables [35,36,37]. Given the importance of understanding the behaviour of *D. stramonium* and its potential spread due to climate change, the main aim of this study was to investigate the germination of *D. stramonium* seeds at different salinity and pH levels and at different temperatures simulating spring and summer conditions. Populations of *D. stramonium* were collected from sites with similar environmental conditions, but under different types of management in order to determine whether the latter also influences germination in the test conditions. The agricultural soil population was collected from Šašinovec, while the undisturbed soil population was collected from Ivanić Grad (henceforth Ivanić).

## 2. Results

### 2.1. Salinity

**Figure 1:**
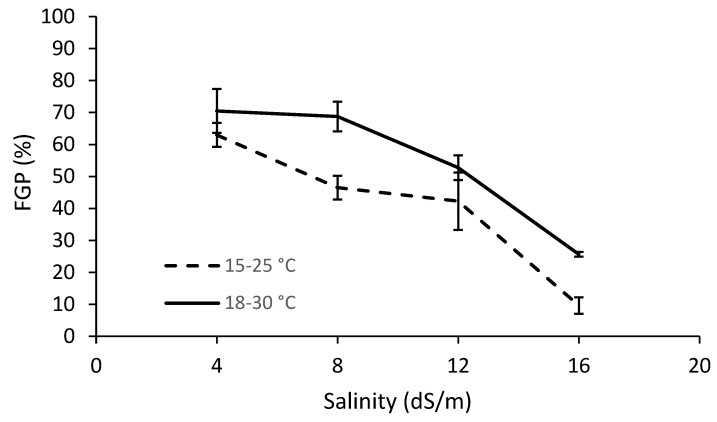
Final germination percentages (FGP) of *D. stramonium* at different temperature ranges and salinity levels (data from the two populations were averaged). Vertical bars represent the standard errors.

**Figure 2:**
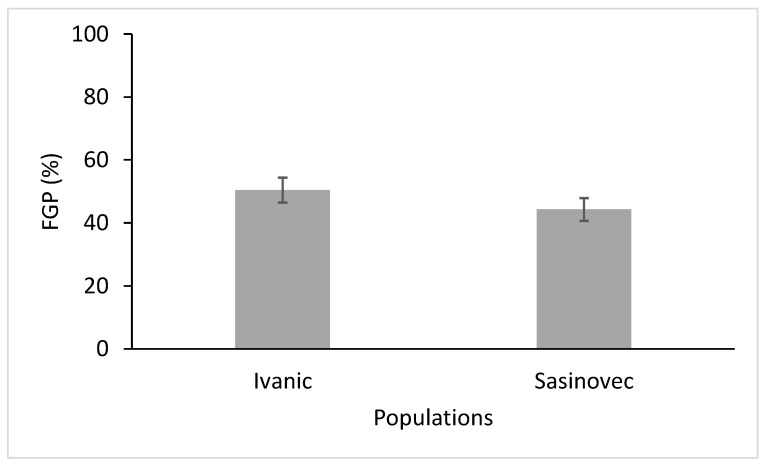
Final germination percentages (FGP) of the two populations of *D. stramonium* (data from the different temperature ranges and salinity levels were averaged). Vertical bars represent the standard errors.

As Figure 1 shows, germination decreases as salinity levels rise and the trend is much more accentuated at the lower temperature range of 15–25 °C than at the higher temperature range of 18–30 °C (the salinity x temperature interaction is statistically significant, as shown in Table 1). The final germination percentage (FGP) of the Ivanić population was generally higher than the Šašinovec population, as can be seen in Figure 2 (the population factor was statistically different for the two sites) (Table 1). Nonetheless, neither the population × salinity nor the population × temperature interaction was statistically significant, while the salinity × population × temperature interaction was at the limit of statistical significance (Table 1).

**Figure 3:**
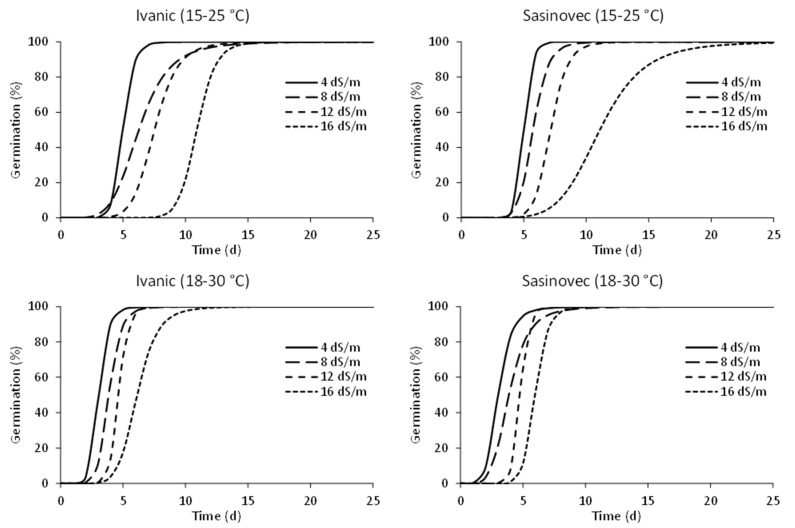
Germination patterns of the two populations of *D. stramonium* in different temperature ranges and salinity levels. The parameters estimated and the measures of goodness of fit are reported in the Appendix A (Table A1).

The germination patterns at different salinity levels (Figure 3) show a gradual forward shift in the time of germination with increasing salinity in both populations and for both temperature regimes, although the shift was more pronounced for the 18–30 °C temperature regime.

### 2.2. pH

**Figure 4:**
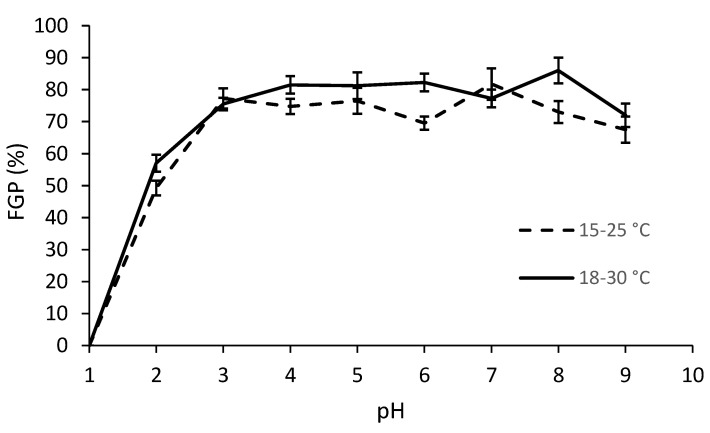
Final germination percentages (FGP) of *D. stramonium* in different temperature ranges and pH levels (data from the two populations were averaged). Vertical bars represent the standard errors.

**Figure 5:**
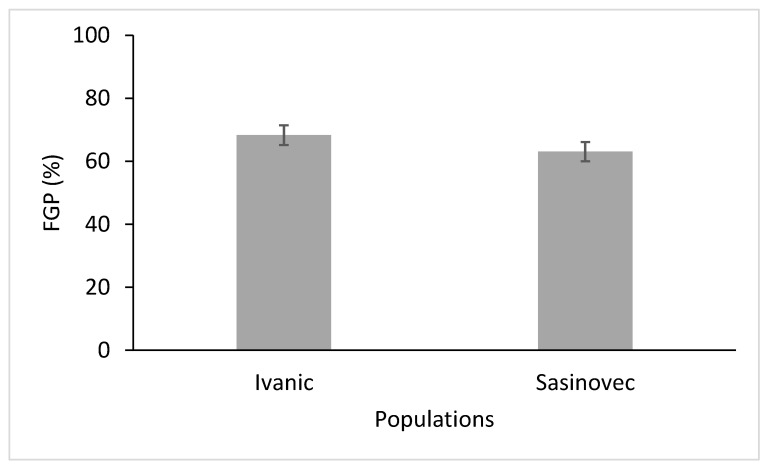
Final germination percentages (FGP) of the two populations of *D. stramonium* (data for the different temperature ranges and pH levels were averaged). Vertical bars represent the standard errors.

Like salinity, pH was also found to affect the final germination of D. stramonium (Table 2). At pH 1, none of the seeds in either population germinated, and at pH 2, a little under half of the seeds germinated, while at higher pH levels, FGP was always around 70–80%. There was also a significant difference in the pH × temperature interactions (Table 2), with a lower FGP in the range of 15–25 °C (Figure 4). Again, the Ivanić population had a higher FGP than the Šašinovec population, as can be seen in Figure 5. However, there was no statistically significant difference in the pH × population x temperature interactions.

**Figure 6:**
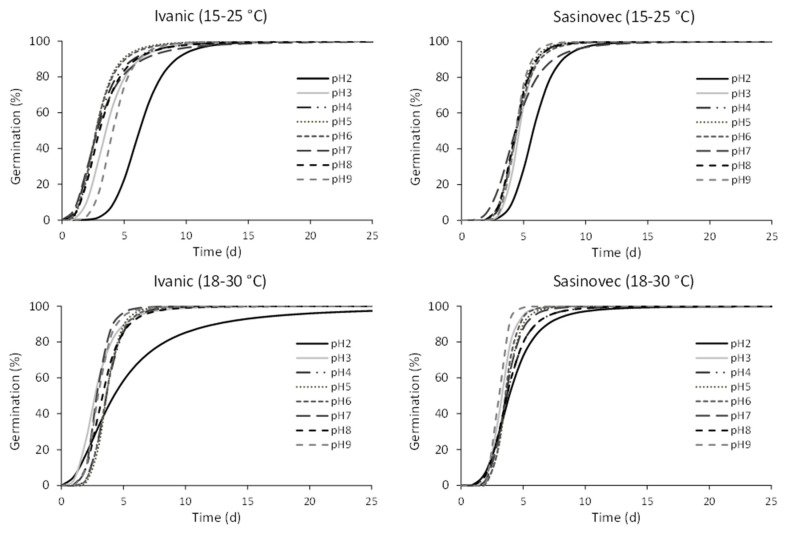
Germination patterns of the two populations of *D. stramonium* in different temperature ranges and pH levels. The parameters estimated and the measures of goodness of fit are given in the Appendix A (Table A2).

The germination patterns at the different pH levels were very similar within the same population and temperature regime, except at pH 2, which differed markedly from the others, especially in the Ivanić population, with a delay in germination (Figure 6).

It is interesting that this species was generally able to germinate at both very low and very high pH levels, except when exposed to pH 1.

## 3. Discussion

There are very little data in the literature on the effects of salinity stress on *D. stramonium* germination. Nevertheless, some of the available data, despite not concerning germination response per se, provides a useful reference for better understanding the results obtained in this work [38,39,40]. Abdel Rahman et al. [39] tested growth and dry mass production at 2.5, 5, 7.5, 10, and 12.5 dS/m plus controls, while Niakan et al. [40] performed similar tests at 2, 4, and 6 dS/m plus controls. In both studies, the researchers noted a reduction in shoot length and the fresh and dry weights of the roots with increasing levels of salinity stress. These results show that salinity tolerance decreases with increasing salinity, which is consistent with the results for germination obtained in the present work. The authors also found increased amounts of alkaloids in the plants exposed to salinity stress, which could be this species’ strategy to survive salinity stress. Besides alkaloids, the accumulation of some amino acids, such as proline, could also be part of this strategy [38,39,40]. Although data are scarce in the literature, there is some information on other species of the Solonaceae family that can tolerate some extreme salinity levels. *Lycium humile*, for example, can tolerate salinity up to 60 dS/m and germinate [41], while some other species, such as *Physalis angulata* and *Physalis philadelphica* var. *immaculata*, can tolerate up to 40 dS/m [42]. Even though *D. stramonium* was not tested under such extreme conditions, given that it belongs to the same family, it cannot be excluded that it might also be tolerant to such high levels of salinity.

As with salinity, there are little or no data in the literature on the germination and growth of *D. stramonium* at different soil pH levels. Nonetheless, some of the available data seem to be in accordance with our findings. Demeyer et al. [43] found no differences in plant growth between pH 5 and pH 7, except for the production of lower amounts of alkaloids in the aerial parts of plants grown at pH 5. Similarly, Saenz-Carbonell et al. [44] found no significant reduction in the root growth of *D. stramonium*, even at a pH lower than 3.5. However, they found that at low pH, there was increased production of alkaloids, especially scopolamine, followed by hyoscyamine. They also noted that the production of large amounts of these alkaloids increased the mean pH level, which therefore needed to be readjusted [44]. This behaviour could explain the tolerance of this species to low pH, which was also observed in the germination tests conducted in our work. Certain other species of the Solonaceae family also exhibit similar resistance to pH levels, especially lower values. Seed emergence of both *P. angulate* and *P. philadephica* var. *immaculata* was around 20% at pH 4 [42], while in some cultivated species of this family, such as *Capiscum annuum*, emergence can be up to 55% at pH 3 [45]. It is interesting that some of the plants used for soil remediation, such as *Paulownia tomentosa*, show little or no germination at pH 4 [46]. Given the results obtained in this experiment, *D. stramonium* could be considered a species for soil remediation.

Regarding the different temperature ranges, several experiments [47,48] have shown that *D. stramonium* germinates better at high or medium-high temperatures and becomes stressed at extremely high or low temperatures. This would explain why the germination percentage in the present study was higher in the higher temperature range. Weeds are also known to be highly variable between populations, especially those that may co-occur with arable crops, such as *D. stramonium* [49,50]. This would explain the differences between the two populations tested in their final germination percentages and patterns while responding similarly to the abiotic stresses to which they were exposed. The similarities in behaviour of the populations also indicate little or no influence of soil management on germination.

## 4. Materials and Methods

### 4.1. Seed Collection

*D. stramonium* seeds were collected in October 2020 from two different sites in mainland Croatia, namely Šašinovec (45°50′59.6″ N 16°09’53.9″ E) and Ivanić Grad (45°69′44.5″, 16°39′81.1″). The seeds were cleaned, dried at room temperature, then stored in a refrigerator (4 °C) until the start of the experiments. The two sites were selected for their relative vicinity to each other and similar pedo-climatic conditions. The mean annual temperature is around 11.6 °C, the mean annual rainfall is around 917 mm, and the soil pH is in the range of 6 to 6.5 [51,52]. The two sites differ in their use, the Šašinovec site being agricultural, while the Ivanić Grad site is undisturbed terrain.

### 4.2. Salinity Test

For the salinity test, four different levels of salinity were used expressed in dS/m (decisiemens/metre): 4, 8, 12, 16; the control was distilled water. These levels are similar to those used by [53]. It was decided to start at 4 dS/m, as this is the level at which the soil is considered saline [54,55], and then go up to 16 dS/m, which is considered highly or extremely saline soil [56,57]. The saline solutions were prepared by adding NaCl to distilled water, and the salinity was determined by measuring the electrical conductivity with a conductivity meter (XS Instruments COND 80, Giorgio Bormac s.r.l, Italy). The seeds were sown in 9 cm-diameter Petri dishes and the saline solution, or distilled water in the case of the controls was added until the filter paper on which the seeds were subsequently placed was fully imbibed. For each salinity level, four replicates of 50 seeds were used under two temperature ranges. The Petri dishes were placed in climate chambers and exposed to a 12 h:12 h light/dark photoperiod at 15–25 °C to simulate spring temperatures and at 18–30 °C to simulate summer temperatures. In each simulation, the lower temperatures were maintained during the 12 h dark period, and the higher temperatures during the 12 h light period. The light was a 75 lm/W neutral white light, the standard used in climatic cells. Germination was monitored every 2–3 days and all germinated seeds were counted and removed. The experiments were considered finished when all the seeds had germinated or after 10 days without germination, as proposed by Baskin and Baskin [58].

### 4.3. pH Test

To assess the effects of pH on germination, nine values of pH were tested, ranging from 1 to 9. For the low pH values, HCl was added until the desired pH was reached; for the high pH values, NaOH was added until the desired pH was reached. All pH values were measured with a WTW 330 pH meter (WTW GmbH, Weilheim, Germany) at the standard temperature of 25 °C, a similar method to that used in [59]. The experiment was conducted in the same way as the salinity test, but using different pH solutions instead of different saline solutions.

### 4.4. Statistical Analyses

To determine whether there were statistically significant differences in the final germination percentages (FGP) between the different salinity and pH levels, temperatures, and populations of *D. stramonium*, a factorial ANOVA was performed. The assumption of normality and homoscedasticity was verified before conducting the analysis. Germination time data were transformed into percentages of germinated seeds and a nonlinear regression analysis was performed using the log-logistic cumulative distribution function:

GP_t_ = 100/1 + e^(s∙(log(t)) − log(t_50_)) (1)

where GP_t_ is the germination percentage at time t, s is the slope at the inflection point, and t_50_ is the inflection point corresponding to the time taken to reach 50% germination. All statistical analyses were performed in the R environment [60].

## 5. Conclusions

This work brings to light some new and interesting information about one of the most important weed species in various agricultural production systems. The information presented here on the germination of *D. stramonium* under salinity, where this species exhibited a high level of tolerance, and pH stress, where a significant reduction occurred only at pH 2, is very important in understanding the behaviour of this species in different soils. Considering that soil salinization and acidification problems will be enhanced by climate change, our findings could also be useful in investigating the consequences of these global changes for the future spread of the species and the problems it could cause for agriculture, especially with regard to weed–crop interactions. Given that the populations tested did not differ greatly in germination, it is possible that this species passes easily from agricultural to undisturbed terrain and vice versa, which could add to its invasive potential. Knowing the tolerance level of *D. stramonium* to different abiotic stresses might help farmers make decisions on the crops or cultivars to be grown in different soils, especially saline or acidic soils, where this species might outcompete some less tolerant crops or crop cultivars. Information on germination could also be helpful for the cultivation of *D. stramonium* for its medicinal properties. With the tolerance to abiotic stresses exhibited, this species might be considered for cultivation in degraded areas becoming an additional source of income. Further experiments are necessary to investigate the response of *D. stramonium* in its early growth stages to these abiotic stresses.

## Figures and Tables

**Table 1:** The effects of salinity, population, and temperature, and their interactions on the final germination percentage of *D. stramonium*.

Factors	*p*-Value
Salinity	0.0000
Population	0.0001
Temperature	0.0000
Salinity: Population	0.2797
Salinity: Temperature	0.0002
Population: Temperature	0.7421
Salinity: Population: Temperature	0.0593

**Table 2:** The effects of pH, population, and temperature and their interactions on the final germination of *D. stramonium*.

Factors	*p*-Value
pH	0.0000
Population	0.0004
Temperature	0.0009
pH: Population	0.2724
pH: Temperature	0.0473
Population: Temperature	0.1859
pH: Population: Temperature	0.9648

## Data Availability

Not applicable.

## Appendix A

**Table A1:** Parameters of the log-logistic function for each population, temperature regime, and salinity level.

Population	Salinity	Parameter	Estimate	SE	*p*-Value	Lower Limit	Upper Limit	r^2^
**Ivanić 15–25 °C**	**4 dS/m**	**s**	−11.41	0.496	0.000	−12.40	−10.41	1.00
		**t_50_**	4.98	0.043	0.000	4.89	5.07	
	**8 dS/m**	**s**	−5.23	0.770	0.000	−6.78	−3.68	0.93
		**t_50_**	6.27	0.190	0.000	5.89	6.65	
	**12 dS/m**	**s**	−8.15	0.847	0.000	−9.85	−6.44	0.98
		**t_50_**	7.52	0.103	0.000	7.31	7.73	
	**16 dS/m**	**s**	−14.60	4.650	0.003	−23.94	−5.27	0.95
		**t_50_**	10.91	0.171	0.000	10.57	11.25	
**Ivanić 18–30 °C**	**4 dS/m**	**s**	−8.07	4.559	0.049	−17.24	1.09	1.00
		**t_50_**	3.05	0.526	0.000	1.99	4.10	
	**8 dS/m**	**s**	−8.23	1.383	0.000	−11.01	−5.45	0.99
		**t_50_**	3.87	0.037	0.000	3.80	3.95	
	**12 dS/m**	**s**	−12.34	0.801	0.000	−13.95	−10.73	1.00
		**t_50_**	4.64	0.049	0.000	4.55	4.74	
	**16 dS/m**	**s**	−7.59	0.870	0.000	−9.33	−5.84	0.98
		**t_50_**	6.13	0.085	0.000	5.96	6.30	
**Šašinovec 15–25 °C**	**4 dS/m**	**s**	−14.88	0.807	0.000	−16.50	−13.26	1.00
		**t_50_**	5.02	0.052	0.000	4.92	5.12	
	**8 dS/m**	**s**	−9.69	0.682	0.000	−11.06	−8.32	1.00
		**t_50_**	5.75	0.032	0.000	5.68	5.81	
	**12 dS/m**	**s**	−10.75	0.853	0.000	−12.46	−9.04	0.99
		**t_50_**	7.15	0.077	0.000	7.00	7.31	
	**16 dS/m**	**s**	−6.29	1.252	0.000	−8.81	−3.78	0.89
		**t_50_**	11.09	0.398	0.000	10.29	11.89	
**Šašinovec 18–30 °C**	**4 dS/m**	**s**	−5.72	0.787	0.000	−7.30	−4.14	1.00
		**t_50_**	3.02	0.122	0.000	2.78	3.27	
	**8 dS/m**	**s**	−5.08	0.502	0.000	−6.08	−4.07	0.98
		**t_50_**	3.88	0.063	0.000	3.75	4.01	
	**12 dS/m**	**s**	−13.80	0.671	0.000	−15.15	−12.45	1.00
		**t_50_**	4.79	0.045	0.000	4.70	4.88	
	**16 dS/m**	**s**	−11.67	0.913	0.000	−13.51	−9.84	1.00
		**t_50_**	5.97	0.018	0.000	5.93	6.00	

**Table A2:** Parameters of the log-logistic function for each population, temperature regime, and pH.

Population	pH	Parameter	Estimate	SE	*p*-Value	Lower Limit	Upper Limit	r^2^
**Ivanić 15−25 °C**	**pH2**	**s**	−5.59	0.379	0.000	−6.35	−4.83	0.98
		**t_50_**	6.22	0.080	0.000	6.06	6.38	
	**pH3**	**s**	−3.79	0.287	0.000	−4.37	−3.22	0.99
		**t_50_**	3.51	0.075	0.000	3.36	3.66	
	**pH4**	**s**	−2.99	0.230	0.000	−3.45	−2.53	0.99
		**t_50_**	2.76	0.110	0.000	2.54	2.98	
	**pH5**	**s**	−3.75	0.554	0.000	−4.85	−2.64	0.99
		**t_50_**	2.73	0.182	0.000	2.36	3.09	
	**pH6**	**s**	−3.49	0.416	0.000	−4.32	−2.66	0.99
		**t_50_**	2.72	0.153	0.000	2.41	3.02	
	**pH7**	**s**	−2.60	0.217	0.000	−3.03	−2.17	0.99
		**t_50_**	2.83	0.135	0.000	2.56	3.10	
	**pH8**	**s**	−3.18	0.20	0.000	−3.59	−2.78	0.99
		**t_50_**	3.01	0.08	0.000	2.84	3.17	
	**pH9**	**s**	−5.29	0.37	0.000	−6.03	−4.54	0.99
		**t_50_**	4.08	0.05	0.000	3.99	4.17	
**Ivanić 18−30 °C**	**pH2**	**s**	−2.04	0.215	0.000	−2.47	−1.61	0.92
		**t_50_**	4.23	0.267	0.000	3.69	4.77	
	**pH3**	**s**	−3.48	0.383	0.000	−4.25	−2.71	0.99
		**t_50_**	2.70	0.142	0.000	2.41	2.98	
	**pH4**	**s**	−5.43	0.318	0.000	−6.06	−4.79	0.99
		**t_50_**	3.60	0.116	0.000	3.37	3.84	
	**pH5**	**s**	−6.61	0.658	0.000	−7.92	−5.29	0.99
		**t_50_**	3.63	0.193	0.000	3.25	4.02	
	**pH6**	**s**	−5.73	0.252	0.000	−6.23	−5.23	1.00
		**t_50_**	3.59	0.087	0.000	3.42	3.77	
	**pH7**	**s**	−5.89	0.227	0.000	−6.34	−5.43	1.00
		**t_50_**	2.86	0.041	0.000	2.78	2.95	
	**pH8**	**s**	−4.26	0.219	0.000	−4.69	−3.82	0.99
		**t_50_**	3.29	0.094	0.000	3.10	3.48	
	**pH9**	**s**	−5.36	0.237	0.000	−5.84	−4.89	1.00
		**t_50_**	2.96	0.054	0.000	2.85	3.06	
**Šašinovec 15−25 °C**	**pH2**	**s**	−6.34	0.738	0.000	−7.82	−4.86	0.96
		**t_50_**	5.77	0.113	0.000	5.54	5.99	
	**pH3**	**s**	−7.77	0.330	0.000	−8.43	−7.11	0.99
		**t_50_**	4.63	0.036	0.000	4.56	4.70	
	**pH4**	**s**	−6.76	0.331	0.000	−7.42	−6.10	0.99
		**t_50_**	4.39	0.033	0.000	4.32	4.46	
	**pH5**	**s**	−7.10	0.280	0.000	−7.66	−6.54	1.00
		**t_50_**	4.34	0.023	0.000	4.29	4.38	
	**pH6**	**s**	−6.07	0.131	0.000	−6.33	−5.81	1.00
		**t_50_**	4.40	0.017	0.000	4.37	4.44	
	**pH7**	**s**	−4.06	0.155	0.000	−4.37	−3.75	0.99
		**t_50_**	4.32	0.042	0.000	4.24	4.41	
	**pH8**	**s**	−6.42	0.311	0.000	−7.04	−5.80	0.99
		**t_50_**	4.32	0.031	0.000	4.25	4.38	
	**pH9**	**s**	−8.47	0.359	0.000	−9.18	−7.75	1.00
		**t_50_**	4.36	0.022	0.000	4.31	4.40	
**Šašinovec 18−30 °C**	**pH2**	**s**	−3.74	0.369	0.000	−4.48	−3.00	0.97
		**t_50_**	3.88	0.103	0.000	3.68	4.09	
	**pH3**	**s**	−7.06	1.032	0.000	−9.12	−4.99	0.98
		**t_50_**	3.28	0.106	0.000	3.06	3.49	
	**pH4**	**s**	−4.50	0.692	0.000	−5.88	−3.11	0.95
		**t_50_**	3.70	0.212	0.000	3.28	4.13	
	**pH5**	**s**	−6.94	1.392	0.000	−9.72	−4.16	0.99
		**t_50_**	3.65	0.239	0.000	3.18	4.13	
	**pH6**	**s**	−7.83	1.313	0.000	−10.45	−5.21	0.99
		**t_50_**	3.55	0.210	0.000	3.13	3.97	
	**pH7**	**s**	−5.88	1.113	0.000	−8.11	−3.66	0.96
		**t_50_**	3.60	0.244	0.000	3.11	4.09	
	**pH8**	**s**	−4.47	0.360	0.000	−5.18	−3.75	0.99
		**t_50_**	3.69	0.112	0.000	3.47	3.91	
	**pH9**	**s**	−9.71	0.716	0.000	−11.14	−8.28	1.00
		**t_50_**	3.04	0.098	0.000	2.85	3.24	
